# Differential temporal expression of milk miRNA during the lactation cycle of the marsupial tammar wallaby (*Macropus eugenii*)

**DOI:** 10.1186/1471-2164-15-1012

**Published:** 2014-11-23

**Authors:** Vengamanaidu Modepalli, Amit Kumar, Lyn A Hinds, Julie A Sharp, Kevin R Nicholas, Christophe Lefevre

**Affiliations:** School of medicine, Deakin University, Pigdons Road, Geelong, Vic Australia; CSIRO Ecosystem Sciences, GPO Box 1700, Canberra, Act 2601 Australia

**Keywords:** Lactation, Marsupials, Micro RNA, Milk, Development, Mammary gland, Exosome

## Abstract

**Background:**

Lactation is a key aspect of mammalian evolution for adaptation of various reproductive strategies along different mammalian lineages. Marsupials, such as tammar wallaby, adopted a short gestation and a relatively long lactation cycle, the newborn is immature at birth and significant development occurs postnatally during lactation. Continuous changes of tammar milk composition may contribute to development and immune protection of pouch young. Here, in order to address the putative contribution of newly identified secretory milk miRNA in these processes, high throughput sequencing of miRNAs collected from tammar milk at different time points of lactation was conducted. A comparative analysis was performed to find distribution of miRNA in milk and blood serum of lactating wallaby.

**Results:**

Results showed that high levels of miRNA secreted in milk and allowed the identification of differentially expressed milk miRNAs during the lactation cycle as putative markers of mammary gland activity and functional candidate signals to assist growth and timed development of the young. Comparative analysis of miRNA distribution in milk and blood serum suggests that milk miRNAs are primarily expressed from mammary gland rather than transferred from maternal circulating blood, likely through a new putative exosomal secretory pathway. In contrast, highly expressed milk miRNAs could be detected at significantly higher levels in neonate blood serum in comparison to adult blood, suggesting milk miRNAs may be absorbed through the gut of the young.

**Conclusion:**

The function of miRNA in mammary gland development and secretory activity has been proposed, but results from the current study also support a differential role of milk miRNA in regulation of development in the pouch young, revealing a new potential molecular communication between mother and young during mammalian lactation.

**Electronic supplementary material:**

The online version of this article (doi:10.1186/1471-2164-15-1012) contains supplementary material, which is available to authorized users.

## Background

MicroRNAs (miRNAs) are small RNAs involved in post-transcriptional regulation of target mRNAs, thereby regulating protein expression levels. miRNAs have crucial roles in regulating a wide range of cellular functions, such as cell differentiation, proliferation and cell death [1, 2]. Recent studies have shown that secretory miRNAs are found in body fluids including breast milk [3], saliva [4], plasma [5] and urine [6]. The presence of circulating or secreted miRNAs in these body fluids has suggested that secretory miRNAs may function in extracellular cell to cell signalling and participate in intercellular regulation of cell function [7]. It has also been reported that secretory miRNAs, and in particular milk miRNA, may be secreted inside small vesicles called exosomes [3]. Exosomes are small lipid-bilayer membrane vesicles, which are released by cells into the extracellular environment and carry various components including peptides, protein, mRNAs and miRNAs [8]. Exosome packaging is considered as a key factor for the stability of secretory miRNAs in the degrading conditions of the extracellular environment [9]. Apart from being secreted in exosomes, milk miRNAs may also be secreted by an alternative mechanism through milk fat globules [10]. Production of milk is a complex process; milk is composed of various bioactive components supporting early development of the neonate [11, 12]. Therefore it is important to fully understand the functional role of miRNA in milk. Expression studies of milk miRNAs have so far been reported in several eutherian species including human [13], bovine [14], pig [15] and goat [16], and have shown that a number of these miRNAs are related to immune regulation, suggesting they may regulate the immune system of sucklings. In addition, miRNAs are known to have important roles in various biological processes, including organogenesis and morphogenesis during fetal development. Gene knockout studies in laboratory models such as zebra fish and mouse have demonstrated that miRNAs are involved in development through post-transcriptional regulation of mRNA expression [17, 18].

In mammals, the change from intrauterine to extrauterine life is a major transition that is exclusively supported by maternal milk provision. Limited development of neonates is observed after a short gestation in primitive mammals like marsupials and monotremes, and milk has a vital role in supporting the development of immature new born during their early postnatal life [19]. During evolution of mammals, marsupials and eutherians have adopted different reproductive strategies which have given rise to a marked difference in maturation at birth [20]. Eutherians, including humans, undergo a long gestation period allowing greater foetal development *in utero* and their composition of milk does not change significantly except for the short period of colostrum production at the onset of lactation [21]. In contrast, marsupials such as the tammar wallaby (*Macropus eugenii*) have a short gestation followed by a long lactation, and the composition of milk changes significantly throughout the lactation cycle to provide factors for growth and development of the immature neonate [12, 20]. As a result of a short gestation and long lactation period, a significant part of development and growth of the marsupial young occurs postnatally during early lactation [22], and signalling factors necessary for development of the neonate may be secreted by the marsupial mammary gland [23]. Mammalian neonates generally receive immune protection from milk (or colostrum) immediately after birth. In marsupials in particular, the newborn, which does not receive any passive immunity during foetal development, lacks a mature immune system and this renders them more vulnerable to pathogenic infections and therefore they are highly dependent on maternal immune source via milk for adequate protection [24, 25].

Studies of the lactation systems of marsupial such as the allied rock wallaby (*Petrogale assimilis*) [26], the north American opossum (*Didelphis Virginiana*) [27] and the tammar wallaby (*M. eugenii*) [12, 28] shown significant changes in their milk composition throughout lactation in marsupials. The tammar wallaby is the one of most studied marsupials and its lactation period is divided into three phases based on the composition of the milk, growth and behaviour of the young (phase 2A, phase 2B and phase 3, while gestation is phase 1) [29]. The tammar wallaby gives birth to a single altricial young after 28 days of gestation (phase 1 of the reproductive cycle). The new born weighs approximately 440 milligrams at birth [30]. During the early phase of lactation (Phase2A, ~100 days), the young is permanently attached to the teat and the mother secretes dilute milk with a low concentration of protein and lipids but a high concentration of complex carbohydrates [31]. In mid-lactation (phase2B, ~day 100 to 200) the young remains in the pouch without being permanently attached to the teat and milk composition remains similar although changes in milk protein composition have been observed [32]. During the final stage of lactation (Phase3, ~day 300–325), the pouch young begins feeding on vegetation but still consumes milk from the mother. During this phase of lactation, the milk becomes rich in protein and lipids with a reduction in the concentration of carbohydrates [33]. The continuous change in the composition of milk proteins has important roles for the development of the pouch young [34] and therefore the tammar provides an exceptional model to correlate milk composition with the defined developmental changes of joeys during lactation, and the identification of new activates in milk associated with molecular signals [35].

The aims of the present study were first to confirm the presence and identify small RNA in marsupial milk, particularly focusing on regulatory miRNA, and to quantitatively profile the dynamics of marsupial milk miRNA expression during the lactation cycle. In addition, this investigation compared miRNA in lactating mothers milk and circulating blood serum in order to investigate the possibility of transfer of blood miRNA into milk. Finally the study evaluated the putative transfer of milk miRNAs from the mother to the circulatory system of the neonate. Overall, the study supports the concept that milk miRNA represent new signalling agents in the molecular relationship between mother and young that has evolved in mammals.

## Results

### Tammar wallaby milk contains RNA and is enriched in miRNA

**Figure 1:**
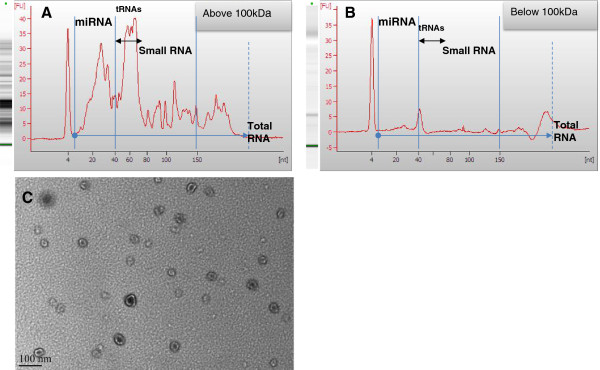
**Tammar milk small RNA profiling on the Bioanalyzer.** RNA from tammar milk exosome co-fraction was analysed on the Bioanalyzer. **(A)** RNAs were mainly detected in the exosome co-fraction, but not in the **(B)** 100 kDa eluted fraction. **(C)** Exosomes vesicles detected by TEM in the exosome fraction.

First, to confirm the presence of RNA in marsupial milk, RNA was purified from tammar wallaby milk collected at day 250. The results confirmed that tammar milk contains significant quantities of total RNA (319.28 μg/ml) and small RNA (sRNA). Profiling on the Bioanalyzer (Figure 1A) showed that a large proportion of milk total RNA was small RNA 83% (265.06 μg/ml) with miRNA representing approximately 66% of total milk RNA (210.65 μg/ml). However, these values may be considered approximate, as the results are obtained from milk that has been through a single freeze-thaw cycle, even though the milk exosomal miRNAs were shown to be partially stable during multiple freeze-thaw cycle [3, 36].

### Tammar wallaby milk miRNA co-purifies with exosome-like vesicles

**Figure 2:**
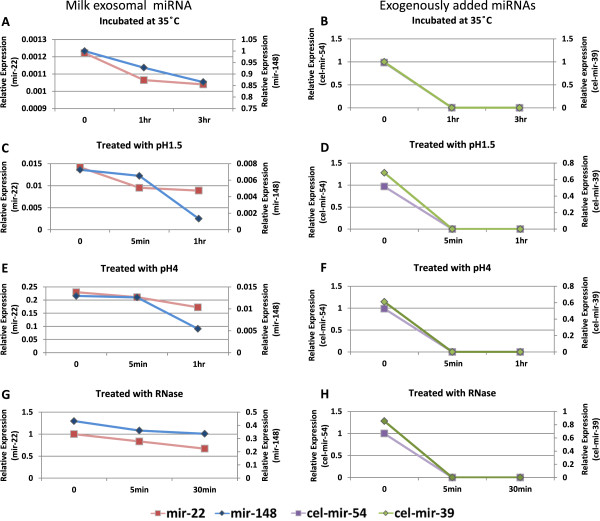
**Stability of tammar milk miRNAs under digestive conditions.** The stability of tammar milk miRNAs tested under various conditions by incubating the milk exosome preparation at **(A & B)** 35°C, **(C & D)** pH1.5, **(E & F)** pH4 and **(G & H)** with RNase. (Left panel) the expression levels of milk exosomal miRNAs mir-22 and mir-148 and, (right panel) the expression level of exogenously added miRNAs cel-mir-54 and cel-mir-39.

To analyse the compartmentalisation of small RNA in milk, tammar milk was fractionated by a series of ultracentrifugation steps followed by filtering as described in methods. Small RNA was shown to remain in the supernatant after ultracentrifugation at 60,000 g for 1 hour and did not pass through a 100 kDa filter (compare Figure 1A and Figure 1B), suggesting that the RNA is likely associated with either high molecular weight complexes or small vesicles. TEM microscopy of the RNA enriched fraction (Figure 1C) revealed that the fraction was also enriched in vesicles of 40 –80 nm diameter, exhibiting similar structure to exosomal vesicles previously reported in milk preparations from eutherian species [13, 36]. These results suggest that RNA is most likely transported within exosomes in tammar milk, although we cannot exclude association with large protein complexes. Further, to analyse the stability of miRNA in the milk exosome fraction under conditions similar to the stomach environment, the exosome preparations were incubated at pH4 and pH1.5, and treated with RNAse (Figure 2). Free synthetic exogenous miRNAs were added to the preparation as a control. The results showed that milk miRNAs were more stable in acidic medium and highly resistant to RNAse treatment in comparison to exogenous miRNAs. This suggests that milk miRNAs in exosome were protected, supporting exosome packaging as a likely factor for their stability.

### Tammar wallaby milk miRNA profiling

**Figure 3:**
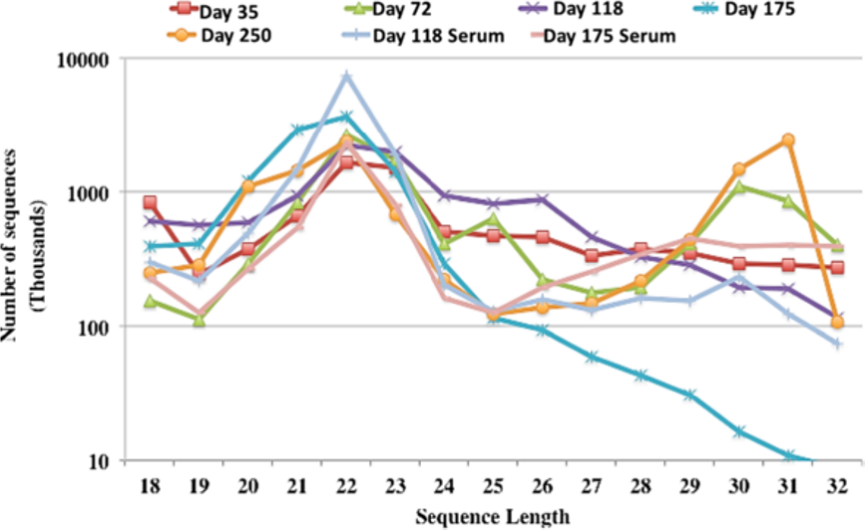
**The distribution of small RNAs of various lengths (18–32 bp) sequences.** Small RNAs were isolated from the tammar milk day 35, day 72, day 118, day 175 and day 250 lactation, and small RNA isolated from tammar lactation mother serum at day 118 and day 175.

In order to characterise the small RNA population of tammar milk, seven small RNA libraries were sequenced on an Illumina hiseq 2000 sequencer. RNA samples were isolated from milk at various stages of lactation and from two additional samples isolated from the blood serum of lactating wallabies also used for milk collection at 118 and 175 days of lactation. Small RNA library sequencing reactions produced from 9 to 13 million raw reads, which were cleaned by removal of low quality reads, reads contaminated with 3 prime and 5 prime adaptor sequences, adaptor only sequences and sequences shorter than 18 nucleotides (Table 1). A total of 4 to 13 million high quality reads were recovered from the libraries. The sequence length distribution of small RNA showed the highest abundance at 22 nt, indicating miRNA-like sequences were the most abundant sequences identified in the majority of samples, representing from 20 to 62% of total RNA sequence reads (Figure 3 and Table 2). Milk samples from Day-72 and phase-3 also showed a high proportion of 30–31 nt sequences mainly representing transfer RNA fragments.

**Table 1:** **Processing of wallaby small RNA libraries**

	Day-35	Day-72	Day-118	Day-175	Day-250	D-118 serum	D-175 serum
**Total reads**	12381908	12134643	13784187	12309932	13709829	14309026	10205531
**High quality**	12335856	12089154	13751725	12280357	13658689	14256868	10180431
**3′adapter**	92168	88219	59397	64848	52910	72041	89445
**Insert**	439041	323346	237928	81011	79542	194066	232346
**5′adapter**	101546	18404	33582	54636	44400	105075	99390
**< 18 nt**	1821675	376879	2041588	1288530	1392025	440562	669988
**polyA**	5	186	311	87	190	6	63
**Clean reads**	9881421	11282120	11378919	10791245	12089622	13445118	9089199

**Table 2:** **MicroRNA analysis using miRanalyzer**

	Day-35	Day-72	Day-118	Day-175	Day-250	Day-118 serum	Day-175 serum
**Total reads**	6781022	7084407	9559993	10596179	6664261	12360382	4776956
**Unique reads**	28968	52333	72537	40811	48558	22639	34143
**% reads aligned to miRNAs**	25.80	50.30	20.80	37.50	62.30	62.30	58.50
**% reads aligned to genes**	0.00%	0.00%	0.00%	0.00%	0.00%	0.00%	0.00%
**miRNA known**	49	90	60	85	90	77	76
**Predicted miRNAs**	45	131	89	101	141	60	55
**Total miRNAs**	94	221	149	186	231	137	131

### Milk miRNA profiles change during the lactation cycle of the tammar wallaby

As discussed earlier, the tammar lactation cycle is divided into three main stages; phase 2A (up to day 100), phase 2B (from day 100–200) and phase 3 (after day 200), with phase 1 corresponding to the period of gestation. While annotated miRNA from serum consistently represented 60% of small RNA sequences, this proportion varied for 20 to 60% of sequences from milk (Table 2). In contrast, milk samples after day 72 of lactation had apparently a slightly more diverse miRNA population than serum despite the fact that milk and serum samples were collected from the same lactating female at day 118 and day 175 (Table 2). Interestingly, this was due to a larger set of newly predicted miRNA from milk. Overall, the number of small RNA sequences, unique reads and miRNAs expressed in milk samples showed some variability due to contamination by tRNA fragments as observed above for Day-72 and phase-3 samples and other experimental factors. Therefore miRNA only sequences annotated by miRanalyzer were collected and proportion-normalised to allow a more relevant comparison of miRNA populations; by dividing the total number of reads annotated for each miRNA by the total number of miRNA reads annotated in respective samples (see Additional file 1).

**Figure 4:**
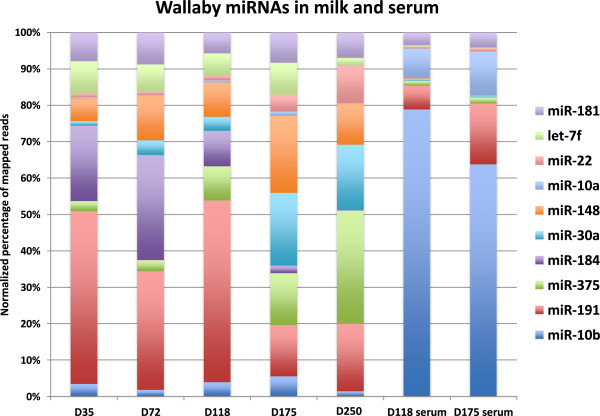
**Wallaby milk miRNA abundance profiles at 5 lactation time points and two serum samples matched to the milk samples.** Bar diagram illustrating the normalized percentage of mapped reads.

**Figure 5:**
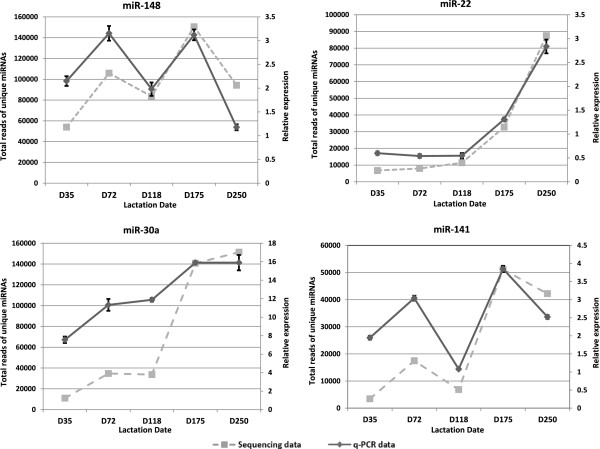
**Tammar milk miRNA expression pattern during lactation.** The expression profiles of highly abundant tammar milk miRNAs were correlated between the q-PCR and sequencing data Values are means ± SD.

The ten most highly abundant miRNAs in milk at five lactation time points and blood serum samples at day 118 and 175 of lactation are summarized in (see Additional file 2). MiR-191 and miR-184 were the most abundant miRNAs in milk from day 35 to day 118. MiR-191 was also present in a very high proportion in the blood serum of mothers. MiR-184 was highly abundant in phase-2A and early phase-2B. Three miRNAs of the let-7 family (7f, 7a and 7i) were also among the top ten miRNAs expressed during the lactation cycle. MiR-181 was consistently in the 6 most abundant miRNAs in all milk and serum samples. MiR-148 showed a gradual increase in proportional abundance from early lactation to the end of phase-2B and was the most highly abundant miRNA at day-175. MiRNA-375 also showed a gradual increase in proportional abundance from early lactation with a peak in phase 3 (Figure 4).Milk miRNA expression data suggested that a number of miRNAs were expressed differentially during lactation. Based on the quantification of sequencing data we selected mir-148, mir-22, mir-30a and mir-141, to confirm this differential temporal expression during the lactation cycle by qRT-PCR (Figure 5). As shown in Figure 4 these miRNAs were also shown to be differentially expressed in milk collected at similar time points of lactation from at least 3 different animals at each time point, excluding milk from the animals used for sequencing. The results correlated well with those from quantitative RNA sequencing, therefore individual PCR confirmed differential temporal expression of all the tested miRNA in milk collected from all other animals.

### Co-expression of wallaby milk miRNA

**Figure 6:**
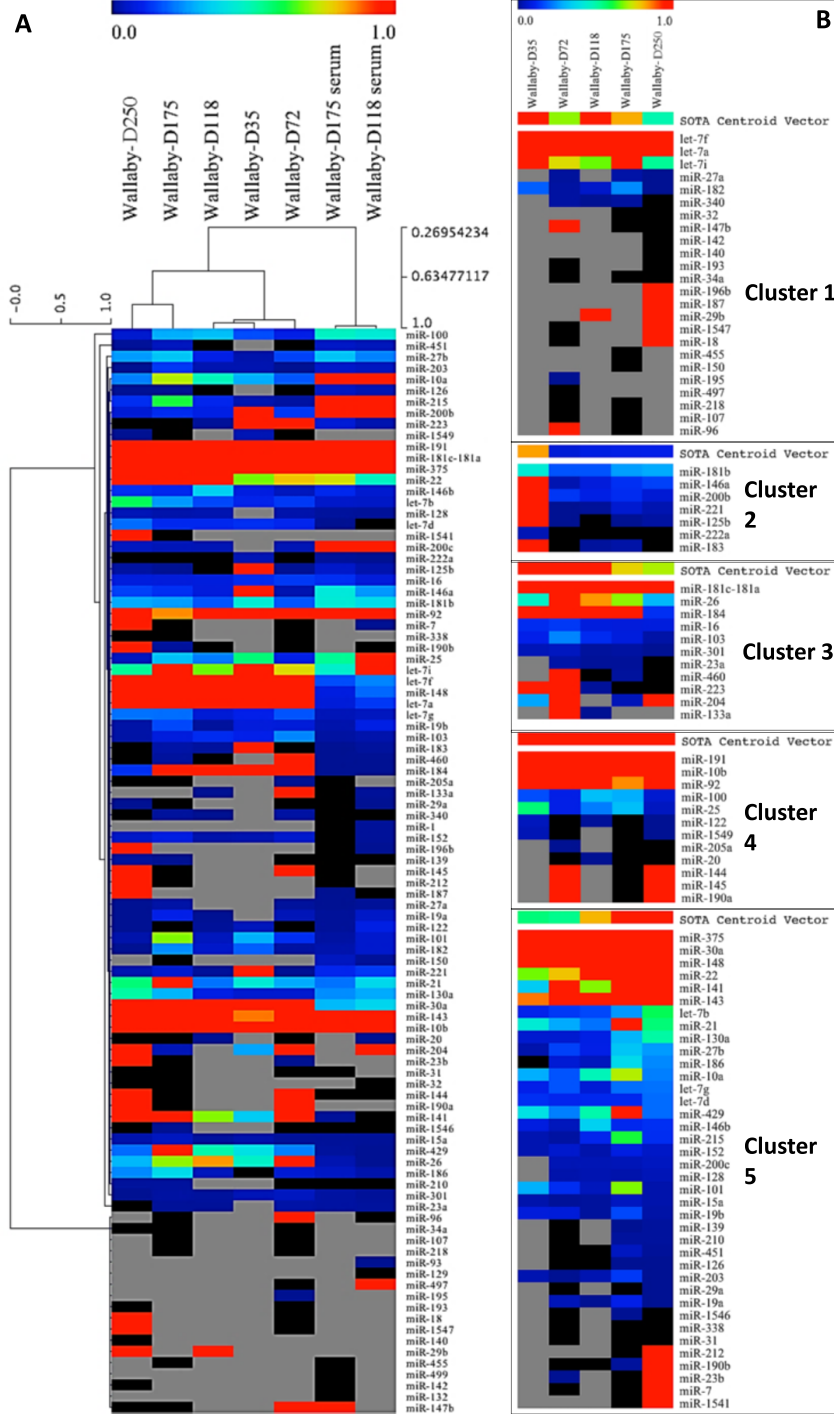
**Clustering of wallaby milk and serum miRNA profiles by Hierarchical Clustering Linkage method: (single linkage Pearson correlation). (6A)** Grey colour in the heat maps denotes the absence of miRNA and black colour indicates miRNAs below 0.001%. **(6B)** Milk miRNA clustering into 5 clusters illustrating individual miRNA expression.

Hierarchical clustering of normalised miRNA proportion profiles showed blood samples were closely grouped together while milk samples were grouped on a different branch of the dendogram (Figure 6A). In order to further identify trends in the co-expression of milk miRNA during the lactation cycle, normalised miRNA quantification data were clustered into 5 groups or clusters by the Self Organizing Tree Algorithm (SOTA) implemented in MeV (Version 4) software, using default settings (Figure 6B). The proportion of miRNAs in each cluster is represented in Table 3. Milk miRNAs in cluster 1 had more variable relative concentration profiles dominated by a biphasic profile with increased proportions in early phase 2A and during phase 2B. This group included let-7 family miRNAs members let-7a, let-7f and let-7i. Cluster 2 was the smallest cluster encompassing only 7 miRNAs. The highest expression values were reported on day-35 with decreasing relative expression thereafter. In cluster 3, miRNAs were highly represented up to day 118 with a gradual decrease in late lactation phases 2B and 3. In cluster 4, miRNAs showed comparable expression across the whole lactation cycle, including miR-191 and mir10b, some of the three most highly expressed miRNA in all samples. Cluster 5 contained the highest number of miRNAs (41%). This cluster consisted mainly of milk miRNAs expressed at all stages of lactation with consistent proportional increase from early to late lactation. The most notable miRNAs in this group were mir-375, mir-30a and mir-148. The proportion of miRNAs in each cluster is represented in Table 3.

**Table 3:** **Proportion of wallaby milk miRNA in clusters from the Self Organizing Tree Algorithm (SOTA) using relative expression levels across 5 time points of lactation cycle**

Cluster	miRNA in Cluster	% of miRNA in Cluster
1	24	26%
2	7	8%
3	11	12%
4	12	13%
5	38	41%

### Comparison of miRNA in wallaby milk and maternal serum

As only minute amount of milk could recovered during the earlier stages of lactation (50 to 300 μl) and to minimize cost, we decided to target more specifically the transitional from phase 2B to phase 3. Lactating mothers at day 118 and day 175 were used to compare miRNA profiles in milk and blood serum of the lactating mother. Overall a similar number of specific miRNAs were identified in milk (86 miRNA) and serum (82 miRNA). However, miRNA composition was widely different in milk and serum (see Additional file 3). Further analysis revealed that 13 miRNAs were milk specific and 9 were serum specific (Table 4). Most of the serum specific miRNAs had low read counts with the highest number of reads mapping to miR-1 (731 reads). In contrast, milk specific miRNAs showed comparatively high expression with, for example, miR-204 represented by 58,142 reads. As shown in Table 4, other miRNAs with over 500 read counts were miR-200b, miR-200c, miR-200a-5p and miR-1549. Interestingly, the genomic locations of miR-200a and miR-200b genes are closely located within a chromosomal cluster. These results suggest co-expression and co-secretion of these closely linked miRNA. The most abundant milk-enriched and serum-enriched miRNAs are listed in Table 5 with their respective normalized expression values. While miR-191 was most highly abundant in milk during the first three time points of lactation (phase 2A), its expression decreased on lactation day-175 (phase 2B) and at the same time, showed a marked increase in serum of mothers. Although the data does not address the specificity and dynamics of circulating miRNA exchanges between maternal blood and milk, and it remains to fully establish if miR-191 is instead no longer transferred from blood into milk from day 175, this observation, together with the general disparity of milk and serum miRNA profiles, suggests that there is no direct correlation between serum and milk miRNA contents. Therefore the data strongly supports the likelihood of local biogenesis of secretory miRNA by the mammary gland rather than transfer from blood into milk.

**Table 4:** **Milk and serum specific miRNAs in lactating wallaby at day 118 and 175 of lactation**

Milk	Reads	Blood Serum	Reads
miR-107	104	miR-1	731
miR-1541	36	miR-129	194
miR-1549	560	miR-132	29
miR-17-3p	41	miR-142	34
miR-190a	86	miR-187	148
miR-200a-5p	515	miR-196b	393
miR-200b	4927	miR-214	10
miR-200c	1948	miR-499	29
miR-204	58142	miR-93	343
miR-218	93		
miR-23b	129		
miR-338	119		
miR-34a	60

**Table 5:** **Highly abundant milk or serum enriched miRNAs**

miRNAs	D118-Milk	D118-Serum	D175-Milk	D175-serum
miR-184*	8.56	0.01	1.45	0.00
miR-148*	8.32	0.20	14.61	0.09
miR-30a*	3.36	0.37	13.67	0.31
let-7f*	5.371	0.238	6.04	0.168
miR-141*	0.684	0.002	4.962	0.01
miR-375*	8.225	1.202	9.782	1.487
miR-191*	43.83	5.77	9.66	14.51
miR-10a**	0.48	7.20	0.71	10.55
miR-10b**	3.42	69.17	3.76	55.40
miR-92**	2.504	2.628	0.849	4.486

miRNAs* are milk enriched and miRNAs** are serum enriched.

### Quantification of milk related miRNAs in pouch young and adult blood

**Figure 7:**
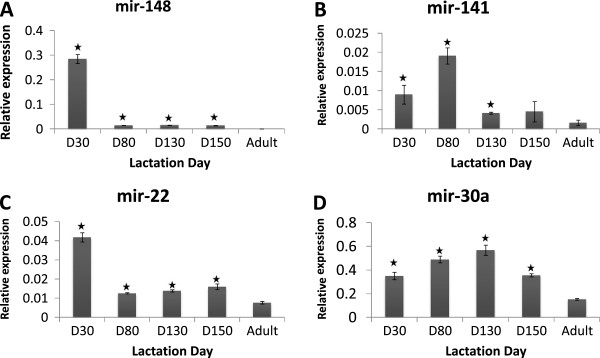
**Quantitative PCR of milk related miRNAs in serum of tammar pouch young during lactation.** Abundant milk miRNAs **(A)** mir-148, **(B)** mir-141, **(C)** mir-22, and **(D)** mir-30a were analysed in tammar pouch young and post-suckling neonate blood serum. The expression was relative to control genes (artificial miRNA spikes cel-mir-39 or cel-mir-54) Values are means ± SD. A significant increase in levels of expression relative to adult (*P* < 0.05).

To analyse the distribution pattern of milk related miRNAs in blood of the pouch young, a subset of miRNAs which were identified above as highly expressed in tammar milk compared to mother serum (mir-148, mir-22, mir-141 and mir-30a) was selected for validation by quantitative PCR. Using the stem-loop RT-PCR method, these miRNAs were quantified in serum of pouch young at lactation day 30, 80, 130, 150 and weaned juvenile (adult), a significant increase in levels of expression relative to adult (*P* < 0.05) (Figure 7). Generally, these miRNAs were in higher proportion in pouch young serum than in the weaned juvenile (adult). The result suggests that highly expressed milk miRNA may transfer to the pouch young serum during early development when the immature gut may allow passive absorption of milk miRNA. However, we cannot exclude independent endogenous biogenesis of miRNAs in pouch young during early development and this would need to be assessed more directly in future experiments.

## Discussion

In recent years, advances in sequencing technologies have allowed the identification of a miRNA population in eutherian milk. In this study for the first time, we have confirmed there are also significant quantities of miRNA present in marsupial milk using the tammar wallaby model.

Milk provides optimal postnatal nutrition for newborn mammals [37]. Milk composition has been investigated in many species [34, 38, 39] and the milk proteins have been considered to make the major contribution to milk bioactivity [34, 40]. However, newly identified milk RNA components, such as miRNA, represent not only potential markers of mammary gland development and activity during the lactation cycle, but also new putative signaling molecules involved in the programming of young development. However, milk miRNA signaling responses in the suckled young have not yet been well defined. We hypothesised that the long lactation period of the wallaby with significant changes in milk composition would allow the characterisation of interesting dynamic patterns of miRNA expression. Cross-fostering experiments have shown that, when a young is fostered onto a mother at a more advanced stage of lactation, an increased rate of growth and of gut development is observed [41]. This indicates that the temporal regulation of milk composition may contribute to the control of development in young marsupials. Therefore secretory milk miRNA may participate in this process and, as a first step towards an understanding of the biogenesis and functional importance of milk miRNA, our study has highlighted the rich diversity and detailed some aspects of the secretion dynamics of the majority of newly identified marsupial milk miRNAs. One limitation of the study is that, as only minute amount of milk could recovered during the earlier stages of lactation (50 to 300 μl) and to minimize cost, single sequencing reactions only were performed at each time point of lactation and it is therefore not possible to estimate biological variation and derive robust statistical models for the analysis of differential expression. To partially compensate for this limitation we have however taken care to normalise the data at the source by pooling milk from different animals (n ≥ 3) before RNA extraction and mainly compared highly expressed miRNA to address the major temporal trend of changes of expression patterns during of the full cycle of lactation rather than conduct differential analysis on pairs of samples.

When all known and co-expressed miRNAs were clustered into 5 groups, the highest number of miRNAs fell in a cluster showing a steady increase in expression from early to late lactation. The study suggests a steady increase in the number of miRNAs and a general quantitative increase of a large group of miRNA during the course of marsupial lactation. This general increase correlates with milk production, phases of mammary gland development during lactation in this organism with a complex lactation system, and the development of organs and improved physiological conditions of the young as it grows in the security of the pouch. This group of miRNA may represent markers of mammary gland activity and putative growth signals. In contrast, the lowest number of miRNAs was in a cluster showing a decrease from early to late lactation indicating miRNA in this cluster are prime candidates for signalling early development of the young.

The serum miRNA profiles were significantly different from all milk samples. Some of the miRNAs (mir-10b, miR-10a, miR-215 and miR-150) were most dominantly expressed in wallaby serum samples and were comparatively low in milk samples. This observation points to a mammary gland biogenesis of miRNAs and secretion in milk, rather than transfer from serum. Although further detailed analysis of specific miRNA is needed to characterise the diffusion of particular sequences, these results also suggest the possible development of milk quality control and diagnostic tests for the detection of infections or pathologies in lactating mammary glands based on the detection of serum specific miRNA in milk.

The overall comparison of serum and milk miRNA populations indicated the abundant milk miRNAs were miR-148, miR-191, mir-141, let-7b, miR-26, let-7d, miR-30a, miR-184, let-7f and miR-375, and the majority of these miRNAs were differentially expressed during lactation (Table 6). Some miRNAs showed a gradual proportional increase from early lactation to late lactation (miR-375, miR-30a, miR-22, let-7b and miR-130a). In contrast, miR-184, let-7f, miR-101 and miR-182 showed a gradual decrease from early lactation to late lactation. In addition, miR-122, mir-222a and miR-1549 were uniquely expressed at day 35 of early lactation and decreased thereafter. Some of these early temporally secreted milk miRNAs are good candidates for developmental signals during early postnatal life.

**Table 6:** **Categories of most abundant miRNAs based on differential expression**

Serum specific	Milk specific	Early lactation (Day 35)	Late lactation Day 250	Gradual decrease	Gradual increases
miR-10b	miR-148	miR-181b	miR-22	miR-191	miR-375
miR-10a	miR-141	miR-222a	miR-200a-3p	miR-184	miR-30a
miR-215	Let-7b	miR-1549		Let-7f	miR-130a
miR-150				miR-101	miR-186
				miR-182	miR-146a
					miR-221
					miR-125b

### Role of miRNA in mammary gland

The mammary gland is a complex organ, which undergoes extensive morphological changes during the lactation cycle, and this remodelling involves cell differentiation, proliferation and apoptosis [42]. Several factors expressed in the microenvironment by epithelium and mesenchyma play key roles in regulating this process, apart from other external hormones and growth factors secreted by reproductive glands [43]. In general, the basic development process of the mammary gland is similar among marsupials (eg tammar wallaby) and eutherians, but events like cell proliferation and differentiation occur for an extended periods in tammars and, simultaneously, the volume of the mammary gland is increased as lactation progresses and larger quantities of milk are secreted [44]. Recent studies have indicated an important role for miRNA in mammary gland development [45, 46] and breast cancer [47]. For example miR-212 and miR-132 are involved in early mammary gland development by regulating epithelial-stromal interactions [45] and the temporal expression of miR-21, miR-205 and miR-200 have potential roles in regulating cell proliferation in the mammary gland during pregnancy [48, 49]. In tammar miR-148 showed a gradual increase in abundance in milk from early lactation to the end of phase-2B and was the most abundant miRNA in wallaby milk at day-175. It is also among the most highly abundant miRNAs in all other mammalian species tested so far [50] (Lefevre, unpublished data). mir-148 has been shown to be important for growth and development of normal tissues [51, 52], and may control growth of mammary epithelial cells. In addition, mir-141, which was also shown to be expressed in tammar milk is involved in the regulation of lactation through STAT5 protein expression [46, 53], as in the mammary gland STAT5 plays a central role in mediating several pathways controlled by lactogenic and galactopoietic hormones [54]. Here, we observed high concentration of miR-141 expression was highly expressed in milk samples compared to serum and expression gradually increased as lactation progressed and milk secretion increased. Similarly, mir-146 has been implicated in the regulation of the innate immune response [55] and the gradual increase in the expression of miR-146 during lactation may reflect its activity in protecting the mammary gland from microbial infection such as mastitis. The let-7b miRNA is generally involved in cell proliferation [56] and also regulates the expression of growth hormone receptors (GHR) [57]. The gradual increase in expression of this miRNA during tammar lactation may reflect mammary gland activity associated with cell proliferation and lipid metabolism as lactation progresses [58]. On other hand mir-181, also consistently abundant in all wallaby milk and serum samples, has been implicated in many cellular processes related to the immune system [59] and also in determination of cell fate and invasion [60]. In summary, all these molecules represent non-invasive candidate markers for physiologic and developmental analysis of the lactating mammary gland. More intriguingly, some of these miRNAs might also function in newborns to control growth.

### Secretory milk miRNA may regulate growth and development of the neonate

Based on miRNA expression patterns during lactation and the identification of milk specific miRNAs, we speculate that some of these miRNAs may represent promising candidates to signal specific development of the pouch young. The role of milk miRNAs is being intensely debated in lactation biology. Variation of miRNA profiles may represent changes in the developmental and metabolic activity of the mammary gland during different stages of lactation and may be used further as biomarkers, or may be specifically secreted by the mammary gland to be transported to the newborn as milk bio-actives [50]. This new putative form of signalling and epigenetic programing between the mother and the neonate may use a sophisticated exosomal transport system. The majority of organs in a new-born tammar wallaby are still functionally immature and their postnatal development relies completely on maternal factors supplied through milk [19]. Milk bioactives include growth factors, peptides, fatty acids and hormones. Like other milk bioactives, milk miRNAs may have a crucial role in development due to their participation in gene regulation. Some of the most abundant milk miRNAs are briefly discussed below in relation to their putative functions during development (Table 7).

**Table 7:** **miRNAs differentially expressed in milk and their function during development**

miRNA	Function	Reference
**miR-148**	Growth and development of normal tissues	[52, 61]
**miR-184**	Development of central nervous system and regulates the balance between the neural stem cell proliferation and differentiation	[62–64]
**let-7b**	Neural stem cell differentiation and proliferation	[63]
**let-7 s**	During embryonic development regulates the timing of terminal differentiation	[65, 66]
**miR-122**	Liver-specific and key role in liver development	[67–69]
**miR-22**	Neural system and erythroid development and maturation	[70, 71]
**miR-375**	Highly expressed in hormone secreting organs (pancreas and pituitary gland) and regulate pancreas organogenesis	[72–74]
**miR-204**	Lens and retinal development	[75, 76]
**miR-30**	Kidney development by regulating *Xenopus pronephros* development	[77]

Three members of the let-7 family (7f, 7a and 7i) were abundant in tammar milk. The let-7 family has been shown to play a significant role during animal development [78]. In tammar milk mir-122 is highly expressed at day35 and is significantly down-regulated afterward, this miRNA has been reported to control liver development and may play a role in liver development of pouch young. Gradually increasing miRNA were mir-22, mir-375, mir-30a, let7b and mir-130a and some of these miRNAs have also been involved in animal development. Mir-375 is expressed in organs linked to hormone secretion such as pancreas and pituitary gland and its expression level increases during pancreas organogenesis [72]. Mice mir-375 knockouts exhibit a decrease in the number of β-cells and an increase of α-cells, leading to hyperglycaemia [73]. Perhaps more significantly, this miRNA has been shown to control the establishment of mucosal immunity in the gut via epithelial-T cell crosstalk, as demonstrated by the greater susceptibility to infection of gut specific knockout mice [79]. The large increase of mir-375 in phase 3 of lactation, when the young starts to exit the pouch and add grass to the diet supports the concept of a milk miRNA contribution to the establishment of mucosal immunity in the young. On the other hand, mir-204 is uniquely expressed during early lactation (phase2A) during which the altricial tammar neonate (0.4 grams at birth) is subject to intense nervous system and eye development [80, 81]. Interestingly, mir-204 regulates lens morphogenesis by controlling the expression of lens placode differentiation gene via the Meis2/Pax6 pathway [75]. Other miRNAs showed a gradual decrease during lactation (mir-184, let-7f, mir-101 and mir-182). mir-184 was highly abundant in tammar milk during early lactation phase-2A until early phase-2B, mir-184 has a significant role in regulating the balance between neural stem cell proliferation and differentiation during development [62, 63]. In marsupial at birth the central nervous system is at early stages of development and the majority of development takes place during early postnatal life [82]. Therefore, the high expression of miR-184 in tammar milk during early lactation may have potential role in maturation of the central nervous system. Further, the mir-30 family is involved in kidney development by regulating the extension of nephron through cell differentiation and proliferation, by targeting the Xlim1/Lhx1 transcription factor as shown in *Xenopus pronephros* development [77]. From the expression data we noticed a peak expression of mir-30 in milk after day130 lactation, this may correlate with nephrogenesis in tammar pouch young as their urinary system starts to undergo maturation and become fully functional during late phase 2B and is not matured before 140 days (20 weeks) after birth [83].

### Transport of milk miRNAs to the pouch young through exosomes

Our results indicate that milk miRNAs are differentially expressed during tammar lactation and most miRNAs are likely to be secreted by the mammary gland. To better understand how these miRNAs were secreted into milk and the potential mechanism of uptake of milk miRNAs by the suckling young, milk was fractionated by ultracentrifugation and size cut-off column filtration. The results showed that the majority of tammar milk miRNA co-purified together with other small RNA in a fraction enriched in exosome-like vesicles. These vesicules ranged from 30 nm to 70 nm in size and appeared round in shape, similar to exosomes reported in milk of eutherians, including bovine [14], porcine [36] and human [84, 85] species. These results suggest that miRNAs in tammar milk are likely to be transported through exosome vesicles and potentially play a role in communication of a diversity of potential molecular signal between cells [86]. Further analysis of these exosomal miRNAs revealed they displayed stability under harsh conditions, indicating that milk miRNAs may successfully be transported to the pouch young without degradation and may survive longer in the gut. Therefore, the presence of exosome-like vesicles in milk suggests that these secretory vesicles may participate in the protective packaging and transport of bioactivities and signals to the neonate and during its subsequent development in the pouch. To address this important issue of a new signalling pathway between mother and young, the digestive system of the young needs to be considered. The digestive system of marsupials is very immature at birth [41, 87] and therefore tammar milk miRNAs may be absorbed through the premature gut system of tammar pouch young and transferred into the blood of the neonate where they could be detected at high concentration. This is possibly in contrast with a majority of eutherians species where the digestive system is far more mature before birth and, soon after birth, the uptake of macromolecules such as immunoglobulins is rapidly ceased [88–90]. The transfer of milk specific miRNAs to the blood circulation of the neonate was detected for a number of highly concentrated milk miRNA quantified in blood samples from pouch young at different stages of development, in contrast to the blood miRNA content of the post suckling adult stage. It is important to consider that a majority of miRNAs may be produced endogenously by the neonate, and therefore this study focused on miRNAs that were shown to be overexpressed at least a thousand fold in milk compare to the mother serum miRNAs, as known from miRNA sequencing. From the qRT-PCR results it was observed that the majority of milk miRNAs were present at high levels in the blood of neonates compared to adult blood serum. Although these indirect results are preliminary and further studies will be required to demonstrate this process of exosome uptake by the gut, the present study suggests that milk miRNAs are transported after packaging into exosomes (rather than freely floating in milk) and that milk miRNAs may be absorbed by the neonate gut system and enter the blood circulation. It is possible that exosomes carry specific surface receptors to attach to the gut cell wall, or that exosomes are digested in the gut where miRNAs are secondarily released along with proteins before absorption by gut cells [91, 92]. Alternatively, exosomal vesicles may diffuse passively through the immature gut of the neonate. Further studies are required to address this issue together with further characterisation of the detailed structure, biogenesis and molecular composition of milk exosomes.

## Conclusions

In this study miRNA populations have been quantified in milk of the marsupial tammar wallaby. We have identified suite of marsupial milk miRNAs and describe the major dynamics of milk miRNA secretion during the complex lactation cycle of the tammar wallaby. In this marsupial model of lactation, pronounced temporal variation patterns of milk miRNA composition have revealed miRNA signatures that may be used to clarify the miRNA-dependant regulation of mammary gland activity as well as the relevance of exosomal transport of the molecular miRNA message towards protection and timing of specific developmental stages in the young. We report poor transfer of miRNA from blood into milk in the mother, but provide evidence toward a likely transfer of milk miRNA into the blood stream of the neonate. Demonstrating the dynamics of milk miRNA profiles across the tammar wallaby lactation cycle has highlighted the value of comparative quantitative transcriptomics to improve our understanding of milk composition and, more importantly, has paved the way to address the true potential of milk miRNA functionality and the full impact of milk consumption on health and wellbeing.

## Methods

### Ethical approval

Tammar wallabies (*M. eugenii*) were maintained in open grassy yards in a breeding colony at Deakin University, Geelong, Victoria (May–August 2012) and CSIRO, Canberra, Australia (June-September 2012). All experimentation was approved by Deakin University and CSIRO Animal Welfare committees.

### Milk collection and isolation of miRNA

Prior to collecting milk, female tammar wallabies were anaesthetised either with an intramuscular injection of 0.2 ml (4 mg/kg body weight) of Zoletil 100^®^ (Virbac, NSW, Australia) or by inhalation anaesthesia (4% Isoflurane under oxygen). To optimize milk collection and to induce milk letdown 0.2 IU of Oxytocin-S^®^ (Intervet, Boxmeer, The Netherlands) was administered intramuscularly. Approximately 300–500 μL of milk was collected from each animal by applying gentle pressure to the mammary glands, and it was stored at -80°C until further analysis. To examine the temporal variation of milk miRNA secretion, milk was obtained from mothers at day 35 (*n* =10), 75 (*n* =5) (Phase 2A) 118 (*n* =4), 175(*n* =4) (Phase 2B) and 250 (*n* =4) (Phase 3) of lactation (some early lactations were interrupted due, in part, to the young failing to reattach to the teat). The milk was thawed on ice and samples collected at particular time point were pooled together. About 1 ml of milk mix was use for RNA isolation. The samples were centrifuged at 5000 xg for 10 min to isolate the fat and cells from skim. Skim milk was centrifuged at 17,000 xg for 20 min to isolate any remaining cells and fat. miRNAs were isolated from skim milk using a commercially available miRNA isolation Kit (mirVana™ miRNA Isolation Kit, Ambion) and the isolation was performed as instructed in the manufacturer’s manual. The miRNA was eluted with 100 μl of elution solution provided in the kit. The samples were stored at -80°C until further analysis.

### RNA sequencing

Sequencing libraries were prepared according to the Solexa Small RNA Sample Prep Protocol. Briefly, the RNA was resolved on 15% polyacrylamide denaturing gels. Gel fragments with a size range of 18–40 nucleotides were excised, and the small RNA fragments were eluted overnight with 0.5 M NaCl at 4°C, and precipitated by ethanol. The small RNA was ligated to 5′ and 3′ RNA adapters with T4 RNA ligase, transcribed into cDNA by Super-Script II Reverse Transcriptase (Invitrogen) and amplified by 15 PCR cycles using Illumina™ small RNA primer set that were annealed to the ends of the adapters. The amplified cDNA products were purified and recovered. Finally, Solexa/Illumina HiSeq sequencing technology was employed to sequence the small RNA samples (BGI, Shenzhen, China). The sequencing data was deposited in the Gene Expression Omnibus (GEO accession GSE58941).

### Blood collection and isolation of miRNA

To compare the miRNA distributions of milk and blood serum of lactating mothers, blood samples were collected from the lateral tail vein of lactating mothers at day 118 (*n* =2) and day 175 (*n* =2). To analyse miRNAs expression in blood of pouch young during the course of development, pouch young at age of day 30 (*n* =6), 80 (*n* =4), 130 (*n* =4) & 150 (*n* =3) and adult (post suckling) (*n* =3) were collected from lactating mothers and the blood samples were collected immediately after sacrifice. The miRNAs from blood serum were extracted using a miRNeasy Serum/Plasma Kit (QIAGEN) as instructed in the kit manual. Milk miRNAs were quantified using qRT-PCR. Two exogenous miRNAs spikes of chemically synthesized cel-miR-39 and cel-miR-54 (miRNA sequences from *Caenorhabditis elegans*) were simultaneously used as control genes.

### Analysis of RNA-seq data

Raw FastQ sequence files from the sequencer were cleaned and adaptors were trimmed using FastQ and FastX programs. Because a complete annotated chromosomal sequence of wallaby is not yet available, the closely related Opossum genome reference and annotations were downloaded from Ensemble (Monodelphis_domestica.BROADO5.67). Chromosomal sequences were indexed using Bowtie2 software and wallaby datasets read mapping was performed against Mondom5 genome assembly using Bowtie2 [93]. Genome alignments were visualized and further investigated in SeqMonk (version 0.24.1). A specialized miRNA annotation tool miRanalyzer was used for the identification of miRNA populations in small RNAs sequence libraries. This tool employs the fast read aligner Bowtie to first align sequences to known miRNAs from miRbase version 19 [94], then to noncoding RNA sequence from Rfam and, finally the remaining sequences are investigated in the context of the genome reference. miRNA target predictions were done on miRNA_Targets database (http://mamsap.it.deakin.edu.au/~amitkuma/mirna_targetsnew/index.html) [95]. Ontology analysis was performed using David Bioinformatics Ontology Database.

### Data accession numbers

The data sets supporting the results of this article are available in the GEO database, accession number for the data set is GSE58941.

### cDNA synthesis and q-PCR for miRNA

Stem-loop primers were used for accurate and sensitive detection of miRNAs [96]. miRNA sequences were retrieved from the miRBase sequence database (http://www.mirbase.org/cgi-bin/browse.pl) to design stem-loop and forward primers. The reverse primer used for RT-PCR was always the universal reverse primer (see Additional file 4). The RT mixture included the combination of RNA (100 ng), 10 mM dNTP, stem-loop primer (1 μM) and nuclease free water up to 15 μl total volume. The mixture was incubated at 65°C for 5 minutes and immediately transferred to ice for 2 minutes. The reaction mix (15 μl) of the first RT synthesis, 5X First stand buffer (4 μl), 0.1 m DTT (2 μ), 0.25 μl RNaseOUT (40 units/μl) and 0.5 μl Superscript III (200 units/μl) was incubated in a thermocycler for 30 minutes at 16°C, followed by 30°C for 30 seconds, 40°C for 30 seconds and 50°C for 1 second for a total of 60 cycles. Reactions were terminated by incubating at 85°C for 5 minutes. Real-time PCR was performed using an SsoFast EvaGreen Supermix (Bio-Rad) and CFX96TM Real-Time PCR Detection System (Bio-Rad). The PCR mixture contained cDNA from RT synthesis, 1 μM forward primer, 1 μM universal reverse primer, and 7 μl SYBER Green master mix and made to 14 μl with nuclease free water. All samples were run as triplicates. The expression levels of target miRNAs were normalized to artificial miRNA Spikes cel-mir-39 (tcaccgggtgtaaatcagcttg) and cel-mir-54 (tacccgtaatcttcataatccgag).

### Exosome isolation from tammar milk

This isolation process was performed at 4°C. The milk samples were thawed on ice and centrifuged at 5000 xg for 10 min to isolate the fat and cells. The samples were then centrifuged at 17,000 xg for 30 min to remove the excess cell debris and fat. Samples were subsequently subjected to ultracentrifugation at 50,000 xg for 60 min to remove casein micelles and collect the whey fraction. The whey fraction was filtered through a 0.4 μm PVDF filter and then a 0.2 μm PVDF filter to remove any further complex particles, and also to allow free flow of the sample through in the subsequent filtration step. The filtrate was loaded on to a 100 kDa filter column (Vivaspin 6, Sartorius AG) and centrifuged at 3900 xg for 90 min. The flowthrough purified milk exosome fraction was collected and stored at -80°C.

### Small RNA profiling on the Bio-analyser

miRNA was isolated from the milk exosome fraction and subjected to bio-analyser profiling to analyse the distribution of small RNA and estimate miRNA concentration using an Agilent 2100 Bioanalyzer and a Small RNA isolation kit (Agilent Technologies Australia). The experiment was performed as directed in the kit manual and 1 μl of the RNA preparation was used for the analysis.

### Electron-microscope (TEM) analysis of exosomes

Purified exosomes from milk samples were analysed using TEM. A drop of sample was placed on parrafilm and fixed with 2.5% gluteraldehyde for 10 minutes at room temperature. A copper grid (Formvar film on 300 mesh Cu Grids) was placed on the drop for 30 min. The sample grid was washed twice with deionized water to remove the excess fixative and stained with Nano-Wan (Nanoprobes). Samples were examined under a JEM-2100 LaB6 Transmission Electron Microscope.

### Stability of milk exosome miRNAs

The stability of milk miRNAs in the exosomal fraction were examined under the following conditions: (A) pH1.5 for 5 and 60 minutes; (B) pH4 for 5 and 60 minutes, the pH was altered by stepwise addition of 1 N HCL aliquots as required and (C) treatment with RNase was for 5 and 30 minutes at 37°C. Two chemically synthesised, free exogenous miRNAs which have no sequence similarity to tammar milk miRNAs (cel-mir-39 and cel-mir-54) were included with the exosomes as a control. The miRNAs were isolated and quantified by q-PCR as described above.

### Statistical analysis

The Q-PCR analysis were represented as mean ± standard error of mean (SEM) and the expression was statistically analysed by applying student *t*-Test. Differential expression was considered significant when P <0.05.

## Electronic supplementary material

Additional file 1: Table S1: Table listing the miRNAs expression in tammar wallaby milk and serum. Normalized miRNA expression of mapped reads in respective milk and serum samples. The empty boxes represent absence and 0 represents low expression of the respective miRNAs. (XLSX 23 KB)

Additional file 2: Table S2: miRNAs found in individual samples of milk and serum from tammar females. (PDF 62 KB)

Additional file 3: Figure S1: Venn diagrams of the distribution of miRNAs found in milk and serum at lactation days 118 and 175. (PDF 53 KB)

Additional file 4: Table S4: Primer sequences used for miRNA quantification by PCR. (PDF 55 KB)
