# Supplementary material for: Differential temporal expression of milk miRNA during the lactation cycle of the marsupial tammar wallaby (Macropus eugenii)
Source: BMC Genomics. 2014 Nov 23;15(1):1012. doi: 10.1186/1471-2164-15-1012 (PMC4247635; doi:10.1186/1471-2164-15-1012)
Supplement: Supplementary file 2 — Additional file 2: Table S2: miRNAs found in individual samples of milk and serum from tammar females. (PDF 62 KB) [file 12864_2014_6694_MOESM2_ESM.pdf]

Additional file 2: miRNAs found in individual samples of milk and serum from tammar females.

**Day-35**	**Day-72**	**Day-118**	**Day-175**	**Day-250**	**D-118 serum**	**D-175 serum**
miR-191	miR-191	miR-191	**miR-148***	**miR-375***	miR-10b	miR-10b
miR-184	miR-184	miR-184	miR-30a	miR-191	miR-10a	miR-191
let-7f	**miR-148***	**miR-148***	**miR-375***	miR-30a	miR-191	miR-10a
miR-181c,a	miR-181c,a	**miR-375***	miR-191	**miR-148***	miR-181c,a	miR-92
**miR-148***	let-7f	let-7f	let-7f	miR-22	miR-92	miR-181c,a
let-7a	miR-30a	miR-181c,a	miR-181c,a	miR-181c,a	miR-215	miR-215
miR-92	**miR-375**	miR-10b	miR-143	miR-141	let-7i	miR-375
miR-10b	let-7a	miR-30a	miR-141	miR-143	miR-143	miR-143
**miR-375**	miR-141	let-7a	miR-10b	let-7f	miR-375	miR-22
let-7i	miR-204	miR-92	let-7a	let-7a	miR-25	miR-25

***MiRNAs mir-148 and miR-375 are highlighted to show the increase in their abundance along the lactation cycle.**
