# Supplementary figures and images for: Differential temporal expression of milk miRNA during the lactation cycle of the marsupial tammar wallaby (Macropus eugenii)
Source: BMC Genomics. 2014 Nov 23;15(1):1012. doi: 10.1186/1471-2164-15-1012 (PMC4247635; doi:10.1186/1471-2164-15-1012)

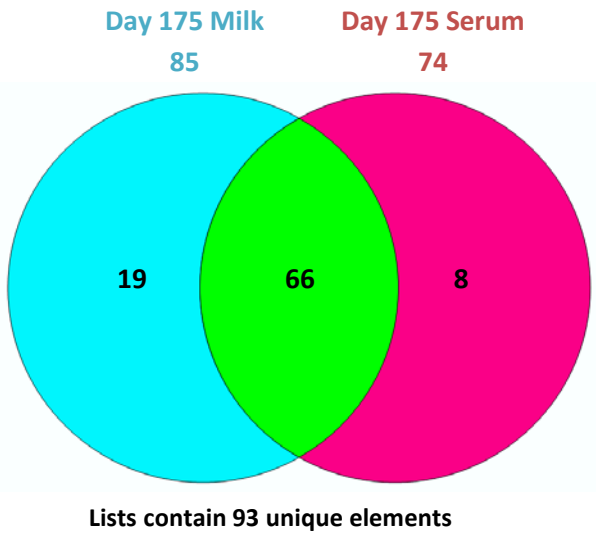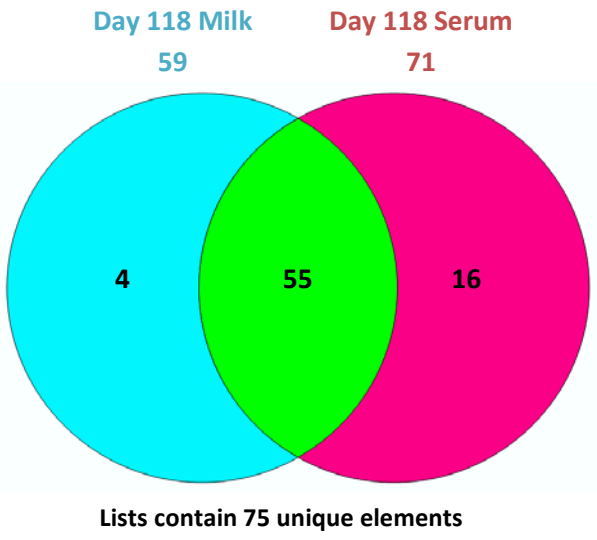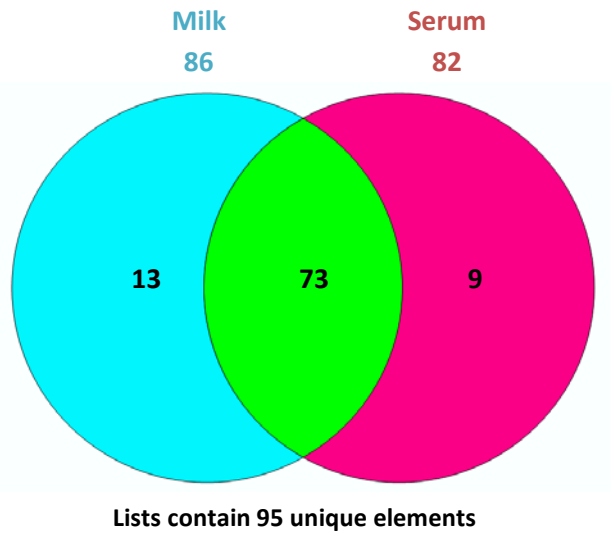

Supplement: Supplementary file 3 — Additional file 3: Figure S1: Venn diagrams of the distribution of miRNAs found in milk and serum at lactation days 118 and 175. (PDF 53 KB) [file 12864_2014_6694_MOESM3_ESM.pdf]
