# Supplementary material for: Differential temporal expression of milk miRNA during the lactation cycle of the marsupial tammar wallaby (Macropus eugenii)
Source: BMC Genomics. 2014 Nov 23;15(1):1012. doi: 10.1186/1471-2164-15-1012 (PMC4247635; doi:10.1186/1471-2164-15-1012)
Supplement: Supplementary file 4 — Additional file 4: Table S4: Primer sequences used for miRNA quantification by PCR. (PDF 55 KB) [file 12864_2014_6694_MOESM4_ESM.pdf]

Additional file 4: Primer sequences used for miRNA quantification by PCR.

miRNAs	Stem-loop primers	Forward primers
**mir148**	GTC GTA TCC AGT GC. GGG TCC GAG GTA TTC GCA CTG GAT ACG AC ACA AAG	CCG GGC TCA GTG CAC TAC AG
**mir22**	GTC GTA TCC AGT GCA GGG TCC GAG GTA TTC GCA CTG GAT ACG AC GCA GTT	CCA GGC AAG CTG CCA GTT G
**mir141**	GTC GTA TCC AGT GCA GGG TCC GAG GTA TTC GCA CTG GAT ACG AC GCA TCT	CGC GCC TAA CAC TGT CTG GTA
**miR30a**	GTC GTA TCC AGT GCA GGG TCC GAG GTA TTC GCA CTG GAT ACG AC CTT CCA	GGC GCG TGT AAA CAT CCT CGA
**miR92**	GTC GTA TCC AGT GCA GGG TCC GAG GTA TTC GCA CTG GAT ACG AC CAG GCC	GCG CTT ATT GCA CTT GTC CCG
**cel-miR-54**	GTC GTA TCC AGT GCA GGG TCC GAG GTA TTC GCA CTG GAT ACG AC CTC GGA	CGC GCG TAC CCG TAA TCT TCA
**cel-miR-39**	GTC GTA TCC AGT GCA GGG TCC GAG GTA TTC GCA CTG GAT ACG AC CAA GCT	GAG CGC TCA CCG GGT GTA
**Universal reverse primer**		CCA GTG CAG GGT CCG AGG TA
